# Predicting clinically promising therapeutic hypotheses using tensor factorization

**DOI:** 10.1186/s12859-019-2664-1

**Published:** 2019-02-08

**Authors:** Jin Yao, Mark R. Hurle, Matthew R. Nelson, Pankaj Agarwal

**Affiliations:** 1 Computational Biology, GSK R&D, 1250 S. Collegeville Road, UP12-200, Collegeville, PA USA; 2 Genetics, GSK R&D, 1250 S. Collegeville Road, UP12-200, Collegeville, PA USA

**Keywords:** Drug target discovery, Clinical trial outcomes, Tensor factorization

## Abstract

**Background:**

Determining which target to pursue is a challenging and error-prone first step in developing a therapeutic treatment for a disease, where missteps are potentially very costly given the long-time frames and high expenses of drug development. With current informatics technology and machine learning algorithms, it is now possible to computationally discover therapeutic hypotheses by predicting clinically promising drug targets based on the evidence associating drug targets with disease indications. We have collected this evidence from Open Targets and additional databases that covers 17 sources of evidence for target-indication association and represented the data as a tensor of 21,437 × 2211 × 17.

**Results:**

As a proof-of-concept, we identified examples of successes and failures of target-indication pairs in clinical trials across 875 targets and 574 disease indications to build a gold-standard data set of 6140 known clinical outcomes. We designed and executed three benchmarking strategies to examine the performance of multiple machine learning models: Logistic Regression, LASSO, Random Forest, Tensor Factorization and Gradient Boosting Machine. With 10-fold cross-validation, tensor factorization achieved AUROC = 0.82 ± 0.02 and AUPRC = 0.71 ± 0.03. Across multiple validation schemes, this was comparable or better than other methods.

**Conclusion:**

In this work, we benchmarked a machine learning technique called tensor factorization for the problem of predicting clinical outcomes of therapeutic hypotheses. Results have shown that this method can achieve equal or better prediction performance compared with a variety of baseline models. We demonstrate one application of the method to predict outcomes of trials on novel indications of approved drug targets. This work can be expanded to targets and indications that have never been clinically tested and proposing novel target-indication hypotheses. Our proposed biologically-motivated cross-validation schemes provide insight into the robustness of the prediction performance. This has significant implications for all future methods that try to address this seminal problem in drug discovery.

**Electronic supplementary material:**

The online version of this article (10.1186/s12859-019-2664-1) contains supplementary material, which is available to authorized users.

## Background

Drug discovery and development often begin with a drug target, through which the drug exerts its therapeutic effect in patients with a certain disease or clinical condition (termed as an indication). A target is a broad term which includes many biological entities such as proteins, genes, and RNA, whose modulation (increase or decrease in activity) can provide a therapeutic benefit to a patient. Although selecting an efficacious drug target is the first and most important step in drug development, more than half of clinical trials still fail due to lack of efficacy, i.e., modulating the target’s activity did not provide a statistically significant benefit to patients [1, 2]. Target selection is critical in drug discovery given the long-time frame and high expense of drug development.

Often drug targets come from research publication where evidence is generated to support a hypothesis that inhibition or activation of a target may result in a therapeutic effect for a specific disease indication. For example, amyloid precursor protein is a target suggested for Alzheimer’s Disease (AD). A piece of important evidence to support this hypothesis is that familial AD patients commonly have genetic mutations in the corresponding gene which lead to the production and deposition in the brain of increased amounts of amyloid beta peptide, a major characteristic of AD [3]. With current informatics technology, it is now possible to construct online repositories that aggregate existing knowledge about the association evidence linking potential targets with disease indications. Open Targets [4] is such a platform that provides drug discovery researchers with multiple evidence types including genetic association, pathways, animal models and drugs, that connect targets with indications for validating potential therapeutic hypotheses. At the same time, these online knowledge repositories are also amenable to computational analysis to discover drug target hypotheses using machine learning.

One major challenge in framing this problem from a machine learning perspective is that there are very few positive examples (0.005% of target-indication hypotheses included in Open Targets have approved drugs). However, any insights gleaned from the limited number of pursued targets may be useful in delivering new medicines with lower attrition rates. In this paper, we collated historical outcomes of clinical trials and determined if these clinical outcomes can be predicted retrospectively using multiple machine learning models built on existing evidence of the targets’ biological association with indications. A successful prediction model provides an understanding of how informative the evidence is for clinical success, and is also capable of generating new target-indication hypotheses with a higher potential of being developed into successful medicines.

Another difficulty in building such a model is that not all biological evidence is available for every pair of target and indication due to reasons such as technological limitation and limited disease coverage. For example, as of June 2017, Open Targets contained 26,122 targets, 9150 diseases with 2,857,732 positive associations from 15 evidence sources. Though Open Targets contains over 2.8 million associations, that is still only 0.08% of the possible combinations covered by this data, suggesting that a great deal of association evidence (99.92%) is still to be determined by biomedical researchers and clinicians. Traditional paradigms of machine learning algorithms, learning a mapping from input features (biological evidence) to output prediction (clinical outcomes), may be inadequate in this context. We explored if tensor factorization is useful in the analysis of this sparse biological dataset.

**Fig. 1:**
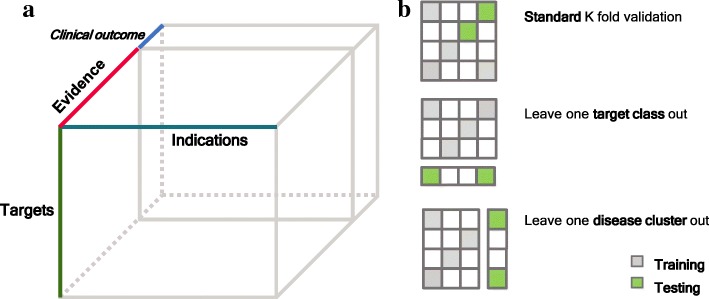
Data representation and model benchmark schematic. **a** Tensor representation of the dataset. The last “slice” matrix represents the clinical outcomes of target-indication pairs. **b** Illustration of three schemes of benchmarking models on predicting clinical outcomes. Each matrix represents the clinical outcomes of targets (rows) and indications (columns). Grey and green cells are target-indications pairs used for training and testing, respectively. Blank cells represent unknown clinical outcomes of target-indication pairs

Tensor extends the concept of a matrix to a multidimensional array where each dimension corresponds to one “axis”, called mode, of a tensor [5]. Data in many applications can be naturally organized into a tensor format. Figure 1a shows a three-mode tensor representing different types of evidence associating targets with disease indications and one extra “slice” represents clinical outcomes. Tensor factorization decomposes a tensor into factor matrices that compactly store information encoded in a tensor and integrate interaction across different modes even when a large portion of entries of a tensor is missing [5]. This technique has a wide range of applications such as in recommendation systems [6], knowledge graph systems [7] and multiple biomedical domains [8].

There are several lines of previous work that are related to this paper. One line of work is focused on answering the question of what makes a good drug target by investigating features of targets that are correlated with clinical successes in the context of genetics [9], tissue mRNA expression [10], human protein interactome [11] and publication trends [12]. Another line of work is focused on disease gene prediction, where the goal is to predict genes mechanistically involved in a given disease [13–17]. Our work is different from these efforts in that we leverage a novel computational method to integrate multiple evidence types and directly assess the models’ performance of predicting clinical outcomes of drug target hypotheses.

In the following sections, we first describe the dataset we have collected and then introduce the basic formulation of matrix factorization, a special form of tensor factorization. Then we explain our selection of a specific algorithm of tensor factorization based on characteristics of our data. We then discuss how we design experiments to benchmark the method against a series of baseline models under three scenarios of drug discovery. We demonstrate that the model can capture known biological mechanisms of human diseases and can identify opportunities of approved drug targets to novel indications.

## Methods

### Data collection and processing

We created a dataset which combined clinical outcomes from the commercial database Pharmaprojects [18] with evidence from Open Targets [4] and other sources (Tables 1, 2). In total, we collected 17 association evidence sources that connect potential targets with disease indications. These 17 association evidence sources can be further grouped into seven evidence types: Genetics (is germline mutation in the target associated with the disease?), somatic mutations (is somatic mutation in the target associated with the disease, typically cancer?), pathways (is the target part of a pathway involved in the disease?), mRNA disease expression (does the target’s expression significantly change in the disease?), mRNA tissue overexpression (is the target’s expression overexpressed in disease-related tissues?), literature (is there association between the target and the indication identified through text mining of scientific literature?) and animal models (does the knockout of the target in animal models manifest phenotypes that are concordant with the human disease?). Besides these 17 association evidence sources, we also collected information about properties of targets from six sources (Table 2) as previous studies have found that successful FDA-approved drugs are enriched with targets with properties that are independent of disease indications [19, 20]. The collected evidence covers the data space of 21,437 targets, 2211 indications, and 17 evidence sources.

**Table 1:** 17 sources of target-indication evidence

Evidence Type	Sources
Animal models	Phenodigm
Genetics	European Variant Archive, Uniprot, Uniprot literature, GWAS catalog, STOPGAP [9]
Somatic mutation	Cancer Gene Census, European Variant Archive somatic
Literature	Europe PMC, TERMITE
mRNA disease expression	Expression Atlas, Internal expression data
mRNA tissue overexpression	GeneLogic, GTEx [49], Human Protein Atlas [50]
Pathways	Reactome, Metabase

Evidence data were obtained from Open Targets [1] except for TERMITE: www.scibite.com/products/termite; GeneLogic: *GeneLogic Division, Ocimum Biosolutions, Inc.,* Internal expression data, and those explicitly referenced

**Table 2:** Six sources of target-only categorical attributes

Attribute Type	Sources (# of categories)
Mutation Tolerance	ExAc_LoF(3), ExAc_Missense (3), RVIS (3), Mouse Protein identity (2)
Other Target Characteristics	Target Location (7), Target Topology (5)

Genes were broken into non-overlapping categories based on available data. Genes were classified as tolerant, intolerant and unclassified based on data from the Exome Aggregation Consortium [51] and the percentile rank of Residual Variation Intolerance Score [52]. Genes were based on the identification of > = 75% protein homology between human and mouse, data downloaded from BioMart [53]. Target Location and Topology were derived from a review of information from Gene Ontology, InterPro, PFAM, and UniProt

For clinical outcome data, if at least one drug asset for a given target-indication pair was identified as successful, then the target-indication pair was classified as Succeeded. Of the remaining target-indication pairs, if at least one asset had a clinical failure then it was classified as a Clinical Failure. Open Targets presents evidence from each individual source as a numerical value for a target-indication pair, with a positive value representing the strength of evidence. To simplify the further collation of target-indication evidence with target-only attributes (Table 2), we converted numerical evidence value into binary values: 1 indicates a positive association, 0 means that there is no association and unknown evidence is represented as null. We encoded categorical data, typically present in target-only attributes, as multiple binary values with each category converted into a binary value, i.e., having the property or not having the property. Here, we analyzed data mapped to the 574 non-cancer indications (a subset of 2211 indications) with at least one clinical outcome and the corresponding 875 targets (a subset of 21,437 targets). Oncology indications were excluded, as studies have observed that features of successful targets for cancer differ from features of successful targets for other indications [21, 22], moreover, cancer trials fail more frequently than trials for other indications. [23]

### Matrix factorization

The clinical outcomes of existing target-indication pairs can be represented in a matrix format as ***R*** ∈ *ℝ*^*M* × *N*^, where the M rows represent targets and the N columns represent indications. ***R***_*ij*_ = 1 if there is at least one drug that modulates target *i* and is marketed for indication *j*. ***R***_*ij*_ = 0 if all the drugs modulating target *i* are reported failed for indication *j* in the clinic (from Phase I to Phase III). For target-indication pairs that have no outcomes in the clinic, the corresponding ***R***_*ij*_ is empty. The goal is to predict clinical outcomes for all possible pairs of targets and indications i.e. fill out the empty ***R***_*ij*_^′^*s*. Thus, we can treat the problem as completing the target-indication matrix of clinical outcomes. Matrix completion problem has been widely studied in the machine learning community in the context of recommendation systems [6, 24]. A famous application is Netflix’s movie recommendation system, where each user has ratings on a small number of movies and the task is to recommend movies for each user based on existing ratings of other users with similar patterns of movie ratings. Matrix factorization is recognized as one of the more successful methods for this task [6, 25, 26]. The method assumes that the true completed matrix is of low rank and can be approximated by a product of two low-dimensional latent factor matrices that represent rows and columns of a matrix in a joint D-dimensional latent space, i.e. ***R*** ≈ ***U***^*T*^***V***, where $\boldsymbol{U}={\left\{{\boldsymbol{u}}_i\right\}}_{i=1}^M\in {\mathbb{R}}^{D\times M}$, $\boldsymbol{V}={\left\{{\boldsymbol{v}}_j\right\}}_{j=1}^N\in {\mathbb{R}}^{D\times N}$ and ***u***_*i*_ ∈ *ℝ*^*D*^, ***v***_*j*_ ∈ *ℝ*^*D*^ are column vectors of ***U*** and ***V***, respectively. The predicted entries in ***R***_*ij*_ is achieved by the inner product of ***u***_*i*_ and ***v***_*j*_. Learning of ***U*** and ***V*** can be formulated as an optimization problem by minimizing the mean squared error between observed and predicted entries. To avoid overfitting, regularization on the latent factor matrices is added to the minimization problem that can be solved by methods such as stochastic gradient descent and alternating least square [6].

### Bayesian tensor factorization

Many matrix-factorization based methods have been proposed for recommendation systems. To choose an appropriate method to predict clinical outcomes, we considered three aspects of our problem. First, some of the evidence is target-indication specific such as human genetic evidence for each disease, and this has been suggested as related to clinical outcome [9]. Second, in our data, there are several target-only attributes independent of indications, such as target protein location, tolerance of mutation. Thus, the chosen method should also take target-only information into consideration. Third, in drug discovery, it is not uncommon that targets or indications that have never been tested in clinical trials. In the case of movie recommendation systems, this corresponds to recommending movies to users who have not rated any movies in the system or recommending new movies that do not have any ratings in the system. The chosen method should be able to handle this situation.

Given these three aspects, we investigated a method based on tensor factorization, called Macau, that is capable of naturally handling all the three aspects in a unified Bayesian framework and was originally used to predict drug-protein interaction [27]. Tensor extends the matrix concept to a multidimensional array, where each dimension corresponds to one mode of a tensor. Our data can be organized into a three-mode tensor: target × indication × evidence $\mathcal{T}\in {\mathbb{R}}^{M\times N\times K},$where one entry *t*_*ijk*_ indicates the association score in *k*^*th*^ evidence between target *i* and indication *j* and one “slice” of the tensor corresponding to one evidence source organized as a matrix. *M*, *N*, *K* are the number of targets, indications and evidence sources, respectively. To predict clinical outcomes, we appended the clinical outcome matrix ***R*** as one extra “slice” to the evidence tensor (Fig. 1a) and factorized the resulting tensor $\mathcal{X}\in {\mathbb{R}}^{M\times N\times \left(K+1\right)}$. Similar to matrix factorization, tensor factorization decomposes a tensor into a series of low-dimensional latent factor matrices where each matrix represents one mode of the tensor. One direct way to decompose a three-mode tensor is to assume that each entry *x*_*ijk*_ can be expressed as the sum of the elementwise product of three low-dimensional vectors: ***u***_*i*_, ***v***_*j*_.and ***e***_*k*_, representing *i*^*th*^ target, *j*^*th*^ indication and *k*^*th*^ evidence (including the clinical outcomes), respectively in a joint latent factor space, i.e. ${x}_{ijk}\approx {\sum}_{d=1}^D{u}_{di}{v}_{dj}{e}_{dk}$, where *D* is the dimensionality of the latent factors. The latent factors can be further organized in three factor matrices: $\boldsymbol{U}={\left\{{\boldsymbol{u}}_i\right\}}_{i=1}^M\in {\mathbb{R}}^{D\times M}$, $\boldsymbol{V}={\left\{{\boldsymbol{v}}_j\right\}}_{j=1}^N\in {\mathbb{R}}^{D\times N}$, $\boldsymbol{E}={\left\{{\boldsymbol{e}}_k\right\}}_{k=1}^{K+1}\in {\mathbb{R}}^{D\times \left(K+1\right)}$ and ***u***_*i*_ ∈ *ℝ*^*D*^, ***v***_*j*_ ∈ *ℝ*^*D*^,***e***_*k*_ ∈ *ℝ*^*D*^ are column vectors of ***U***, ***V*** and ***E***, respectively. Here the ***e***_*K* + 1_ column of ***E*** corresponds with the latent factor of the clinical outcome. The prediction of any entry *x*_*ijk*_ of the tensor can be achieved by the sum of the elementwise product of the low-dimensional vectors of target ***u***_*i*_, indication ***v***_*j*_and evidence ***e***_*k*_. Since the factorized tensor included the clinical outcome matrix, we can use the low-dimensional vector corresponding to the clinical outcome to perform prediction, i.e. the predicted outcome of modulating target *i* for the treatment of indication *j* is **1**^***T***^ (***u***_*i*_ ∘ ***v***_*j*_ ∘ ***e***_*K* + 1_), where **1** is an all one vector and ∘ is the elementwise product.

The specified tensor factorization method we chose is based on Bayesian probabilistic modeling, which assumes each observed cell of the tensor $\mathbf{\mathcal{X}}$ is a random variable following a normal distribution, ${x}_{ijk}\sim \mathcal{N}\left({\mathbf{1}}^{\boldsymbol{T}}\ \left({\boldsymbol{u}}_i\circ {\boldsymbol{v}}_j\circ {\boldsymbol{e}}_k\right),{\alpha}^{-1}\right)$, where *α* is the precison of the normal distribution. In this model, the mean of the normal distribution is determined by the three low-dimensional latent factors: ***u***_*i*_, ***v***_*j*_.and ***e***_*k*_. Each latent factor is assumed to have a Gaussian prior with a Gaussian-Wishart hyper prior placed on its hyperparameters: ${\boldsymbol{u}}_i\sim \mathcal{N}\left({\boldsymbol{\mu}}_{target}+{\mathbf{B}}^{\boldsymbol{T}}{\boldsymbol{g}}_i,{\Lambda}_{target}^{-1}\right)$, ${\boldsymbol{v}}_j\sim \mathcal{N}\left({\boldsymbol{\mu}}_{indication},{\Lambda}_{target}^{-1}\right)$ and ${\boldsymbol{e}}_k\sim \mathcal{N}\left({\boldsymbol{\mu}}_{evidence},{\Lambda}_{target}^{-1}\right)$, where **Β** ∈ *ℝ*^*G* × *D*^ linearly projects the target-only attributes ***g***_*i*_ ∈ *ℝ*^*G*^ into the latent space, providing prediction ability to targets that do not have any observed clinical outcomes. The inference of model parameters is carried out by sampling from the posterior of the model parameters by Markov Chain Monte Carlo (MCMC) technique, except for *α*, which is set to 1 by default and the number of latent factors *D*, which is determined by a heuristic approach (see Additional file 1). Specifically, we used the Julia implementation of the method [28] and followed a common practice of MCMC inference where we “burn-in” samples generated in the beginning and collect samples after that to approximate posterior distribution over model parameters [29]. In our case, the first 500 samples were discarded and the posterior distribution over parameters were estimated using 300 samples after the “burn-in” process. The predictive distribution is approximated from the 300 samples of the model parameters and the average over samples is used to make predictions. Generally, we did not observe further improvement on prediction performance if we let the chain run longer.

### Model benchmark experiments

We performed a nested cross-validation experiment to evaluate the method in three different scenarios (Fig. 1b). In each experiment, we divided the target-indication pairs with clinical outcomes (6140) into K folds and tested the prediction results on a held-out (one of the K) fold using a model trained with the rest (K-1) of folds. This is the outer loop for the cross-validation. In the inner loop, we determine the model parameters using five-fold cross-validation. In the first experiment, we did a standard ten-fold cross-validation in the outer loop, where each fold is randomly determined but retains the same fraction of successes. In drug discovery, we know that certain sub-classes of targets and indications have different properties. In order to, assess if the model can be generalized to sub-classes of targets and indications different from those used in the training stage, we devised two other cross-validation experiments where each time the clinical outcomes of one pre-defined group of targets (indications) are left out as the test set. Specifically, for the second experiment, i.e. leave-one-target-group-out, we used the grouping defined by the Target Class (See Table S1 in Additional file  1). A given target is assigned to one of ten target classes (thus K = 10) based on the target’s protein family retrieved from ChEMBL hierarchical target classification system [30]. For the third experiment, i.e. leave-one-indication-group-out, we defined eight indication groups (thus K = 8) by de novo clustering indications based on the similarity of the indications in terms of their relative positions in MeSH (Medical Subject Heading) hierarchical tree and co-occurrence frequency in the literature (see Table S2 in Additional file 1).

### Baseline models

For comparison purposes, we also ran the cross-validation experiments using four additional machine learning models. For these models, each target-indication pair is treated as a data point and the corresponding 17 association evidence and six target-only attributes are treated as its features. As the target-only attributes are not directly linked with specific indications, for each target, we duplicated its feature values across all indications. The task is being cast as a binary classification problem. To allow these four models to handle missing values, we treated the association scores as categorical variables with three categories: no association (0), positive association (1) and unknown (missing) association. Each categorical variable is then encoded as two binary variables (also called one-hot encoding). The four models that we tested are:

1. Logistic Regression (LR), a simple linear model.
2. LASSO [31]***,*** which is a generalized linear model with L1 regularization implemented in the glmnet R package where the regularization parameters were determined using cross-validation.
3. Random Forest (RF), an ensemble model of decision trees where the parameters are determined by the Out-of-Bag error estimate using the *tuneRF* function in *randomForest* R package.
4. Gradient Boosting Machine (GBM) [32], a boosting method which is implemented in *xgboost* [33] where we tuned the following parameters using cross-validation: the feature shrinkage rate, maximum depth of a tree, subsample ratio of features and number of iterations.
5. Matrix Factorization (MF): We also included another baseline model where we *only* used the clinical outcome matrix and applied matrix factorization to complete the matrix for prediction. Specifically, we used a nuclear norm regularized matrix factorization method that is implemented in the *softimpute* [34] R package and the regularization parameter is determined through cross-validation.

### Performance metrics

We used two metrics to measure the prediction performance of the evaluated methods: area under receiver operator curve (AUROC) and area under precision-recall curve. (AUPRC). The AUROC measures the probability of a model ranking a randomly chosen positive example higher than a randomly chosen negative example and is commonly used in assessing the performance of models for binary classification tasks. AUROC treats positive and negative examples equally, this metric is of limited value when the number of positive examples is relatively low. Given the low success rate in drug development, we chose AUPRC as the primary evaluation metric as it focuses on the performance of positive examples. Here the precision is the proportion of correctly predicted positives out of all predicted positives and recall is the proportion of correctly predicted positives out of all positives.

## Results

### Model benchmark results

**Fig. 2:**
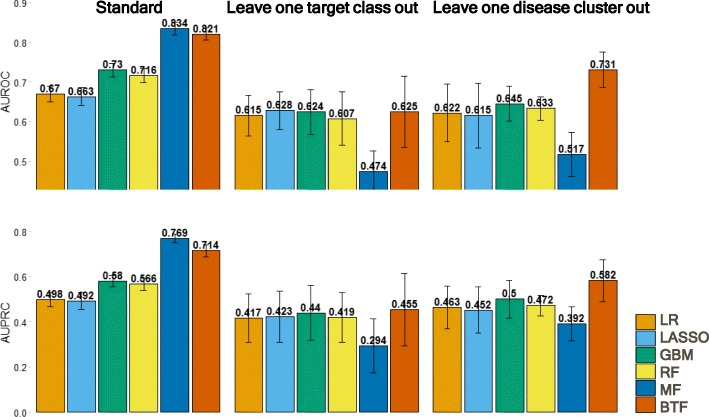
Benchmark performance of models. Prediction performance comparison in three benchmark schemes in terms of Area Under Receiving Operation Curve (AUROC, Top) and Area Under Precision Recall Curve (AUPRC, Bottom). Error bars are calculated from cross-validation (LR: Logistic Regression; GBM: Gradient Boosting Machine; RF: Random Forest; MF: Matrix Factorization; BTF: Bayesian Tensor Factorization)

We performed a standard cross-validation experiment to benchmark various types of machine learning models (Fig. 1b, panel 1). The best model is the matrix factorization (MF) model (AUROC = 0.83 ± 0.02, AUPRC = 0.77 ± 0.02) (Fig. 2), which only factorizes the clinical outcomes matrix without considering any other evidence in the dataset. Due to the highly-correlated structure within the clinical outcomes of target-indication pairs, the standard way of randomly splitting them into training and test sets may overestimate the predictability of clinical outcomes. This may explain the high performance of MF; knowing which targets have succeeded against which indications in the training data may provide enough information to predict the outcome status of new indications for these targets. Many drug targets are from the same gene family, and in a random training-test split, it is likely that targets within the same gene family are split across training and test set, though the same drug may bind to multiple members in each set. This leads to an overestimate of prediction accuracy for truly independent and novel targets. A similar effect may relate indications with different subtypes, as drug targets are often tried against many closely related diseases.

To mitigate this problem and obtain an unbiased estimation of predictive capacity, we designed two benchmark experiments, where a group of similar targets **(**Fig. 1b, panels 2 and 3**)** or indications is held out as a test set and models trained on the other target or indication groups, respectively, are evaluated against the held-out set. We categorized targets into ten target classes largely derived from the ChEMBL hierarchical target classification system [30], and grouped indications into eight clusters that are based on MeSH hierarchy and co-occurrence frequency in the literature (see Additional file 1). In the leave-one-target-class-out cross-validation experiment (Fig. 2), the performance of MF decreases dramatically as there is no information in the training set to predict the clinical outcomes of the held-out target class. All the other methods perform similarly and the overall performance is not as good as in the standard cross-validation setting. This implies that it is difficult to predict candidate indications for targets that have not been assessed in clinical trials. In the leave one disease cluster out validation experiment, the performance of MF again dropped below that of the other methods as there is no information about clinical outcomes of the held-out disease clusters in the training step.

However, the Bayesian tensor factorization (BTF) model scored as the best model in the disease group benchmark (AUROC = 0.73 ± 0.05, AUPRC = 0.58 ± 0.09) and the second to best model in standard cross-validation (AUROC = 0.82 ± 0.02, AUPRC = 0.71 ± 0.03). It is counter-intuitive that BTF does not out-perform the MF method in the standard cross-validation case, as it incorporated more data. MF approach may be taking maximum advantage of the highly-related nature of the outcomes, given the poor performance of MF in the target class and disease group benchmarks. MF also only needs to learn latent factors to explain the clinical outcomes, while BTF needs to learn latent factors to explain the clinical outcomes and all the evidence as well, which is inherently a more difficult task.

In general, the performance of models that explicitly use evidence as predictors did not vary too much across three validation settings. Among these models, ensemble-based methods (RF and GBM) worked slightly better than linear model-based methods (LR and LASSO). Although MF performed relatively well in the standard validation case, its performance was inconsistent among validation settings. BTF combined both evidence and inter-relationship among targets and indications and performed consistently well in all three validation scenarios. In addition to AUROC and AUPRC, we also evaluated performance using F-score, precision@30, and recall@30 (see Additional file 4), but the comparison across methods was not affected.

### Leave one out experiments

**Fig. 3:**
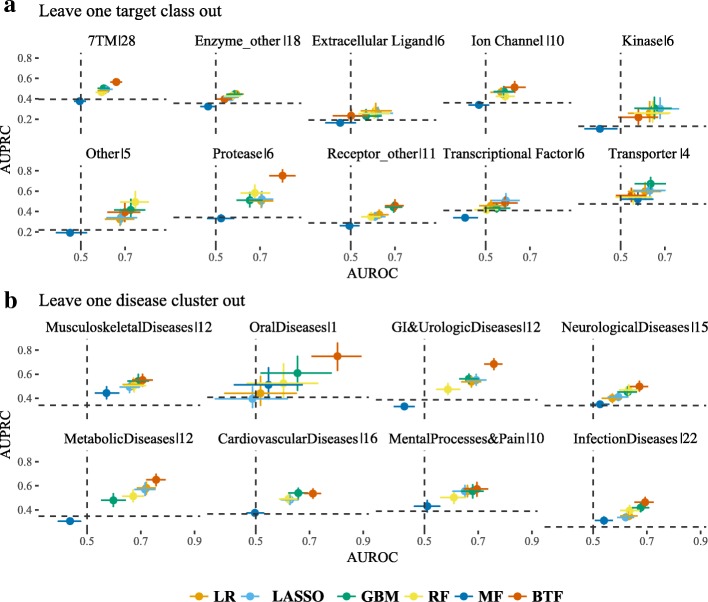
Benchmark performance of leave one out experiments. Model performance on predicting clinical outcomes of target classes (**a**) and disease clusters (**b**) in the leave-one-out experiments in terms of Area Under Receiving Operation Curve (AUROC, x-axis) and Area Under Precision Recall Curve (AUPRC, y-axis). 95% confidence interval is calculated using 1000 bootstraps. Dotted lines mark the AUROC (vertical) and AUPRC (horizontal) of a random guess, which is 0.5 and the fraction of positives in the testing set, respectively. The percentage of target-indication pairs in each held-out set is listed after the pipe symbol (|) in the titles. (LR: Logistic Regression; GBM: Gradient Boosting Machine; RF: Random Forest; MF: Matrix Factorization; BTF: Bayesian Tensor Factorization)

One advantage of this leave one target/disease group out validation scheme is that we can assess how trained models can be generalized to groups of targets/diseases that the models have never trained on before. Figure 3 shows the prediction performance of the six models on the held-out target classes (Fig. 3a) and disease clusters (Fig. 3b). In the leave-one-target-class-out case, the prediction performance averaged over the six models varies between target classes (AUPRC ranges from 0.24 to 0.58; AUROC ranges from 0.53 to 0.68). Specifically, we notice that the models perform consistently poorly for transcriptional factor targets and miscellaneous enzymes, which implies that these target classes are quite different from the other target classes. On the other hand, most models perform relatively well in protease targets. We note the performance is consistent among models within each target class, but this low variability is not repeated in the leave-one-disease-cluster-out case, where the prediction performance shows higher variability among disease clusters. For example, the BTF model performs better than the other models in the metabolic, GI and urologic and oral disease clusters, and performs as well as any other model in the other disease clusters.

### Latent factors capture disease relationship within three disease areas

**Fig. 4:**
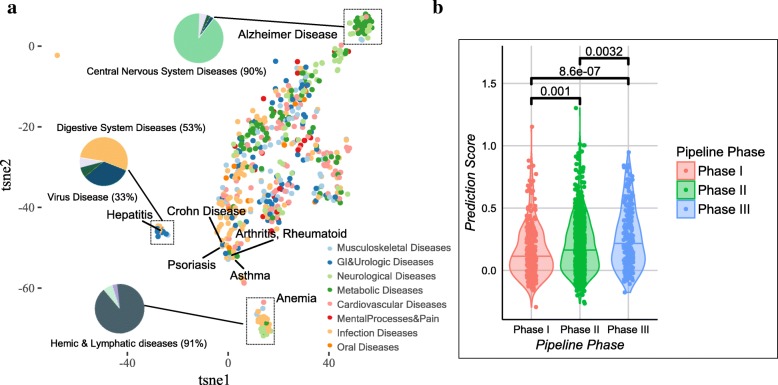
Validation of BTF model prediction. **a** t-SNE visualization of indications based on the latent factors learned in BTF model. Each dot represents one indication and the size of the dot is proportional to the number of targets that have been clinically failed. The inserted pie charts show diseases composition of representative clusters of indications in the 2D visualization. 2D embedding was obtained by using perplexity = 30 in t-SNE and the visualization is consistent using different perplexity values in the range from 10 to 50. **b** BTF prediction scores of target-indication pairs in Phase I-III clinical trials. The numbers are *P*-values (Wilcoxon rank sum tests) from comparing prediction scores of target-indication pairs between any two phases

After benchmarking the performance of the BTF model in the cross-validation experiments, we fitted the model to the whole dataset. We chose 11 latent factors (see Additional file 1). Before using the fitted BTF model to make any predictions, we explored whether the latent factors learned from the model are biologically meaningful so that we can increase our trust in the prediction made by the model. To do so, we reduced the 11 latent factors to two dimensions using t-SNE [35] to visualize how indications are distributed and examined whether the learned latent factors can capture inter-relationship among indications. t-SNE is a dimension reduction technique used to visualize high-dimensional dataset where similar points in high dimensional space are transformed to neighboring points in a low dimensional space and dissimilar points are transformed to distant points in the low dimensional embedding. Figure 4a shows the two-dimensional t-SNE embedding of the 574 indications with at least one clinical outcome, where three distinct clusters are present on the map. We further checked the MeSH annotations of the diseases in each cluster and found that the three clusters are enriched with three distinct disease categories including Central nervous system diseases, Digestive system diseases, and Hemic & lymphatic diseases, respectively. Interestingly, auto-immune diseases, such as rheumatoid arthritis, asthma, psoriasis and Crohn’s disease that manifest in different organs are localized in the same neighboring area on the map. This implies that latent factors are able to capture the intrinsic relationships of diseases within these disease areas. For the rest of the diseases, we did not observe distinct clustering patterns using t-SNE. This could either be because the latent factors are not rich enough to capture the relationship among these diseases or these diseases are inherently interconnected by sharing similar pathological mechanisms [36].

### Prediction scores of target-indication pairs under clinical trials

To validate the prediction made by the BTF model, we chose 1246 novel target-indication pairs that were in clinical trials (Phase I-III) at the time when we collected the data (May 2016), and thus did not have clinical outcome readouts. We compared the prediction scores generated by the BTF model on these target-indication pairs and noticed that the prediction scores of later phase pairs are significantly higher than those of earlier phase pairs (Fig. 4b), which recapitulates the observation that drugs in later phases on average have a higher likelihood of approval [37]. Since we did not include phase information of these target-indication pairs when training the model, these pairs serve as an independent test set and the results increase our confidence in the predictions of the model.

Next, we conducted a literature search on the top 63 hypotheses of the 1246 pairs based on a prediction score threshold, which corresponds with 0.8 precision and 0.27 recall in the standard cross-validation experiment. We list 15 of these 63 hypotheses along with a relevant literature reference in Table 3; the complete list of 63 can be found in Additional file 1.

**Table 3:** High Scoring Pairs of Interest from TF Model

Target	High Scoring Indication in Clinical Pipeline (Phase*)	PubmedID	Related Approved Indication (Total Approved Indications)
ABCC8	Glucose Intolerance (III)	23903354	Diabetes Mellitus (1)
ADRB1	Cachexia (II)	20426789	Ischemia (13)
ADRB2	Hypoglycemia (I)	22013013	Glaucoma (15)
ADRB2	Myocardial Infarction (III)	26692153	Heart Failure (15)
AGTR1	Hypercholesterolemia (III)	12117739	Hyperlipidemias (7)
CYP3A4	Hepatitis C (II)	20938912	HIV Infections (1)
IL2	Behcet Syndrome (II)	26654556	Graft Rejection (1)
IL6	Waldenstrom Macroglobulinemia (I)	26238488	Giant Lymph Node Hyperplasia (1)
IL6	Arthritis, Psoriatic (II)	27789987	Giant Lymph Node Hyperplasia (1)
OPRM1	Schizophrenia (III)	27397309	Migraine Disorders (22)
RYR1	Muscular Dystrophy, Duchenne (I)	26793121	Malignant Hyperthermia (1)
SERPINC1	Hemophilia (II)	27099538	Blood Coagulation Disorders (16)
TNFSF11	Hypercalcemia (II)	27904108	Osteoporosis (1)
VDR	Alopecia (I)	27932380	Keratosis (9)
VDR	Cachexia (I)	22497530	Chronic Kidney Failure (9)

New indications of approved targets in clinical trials (Phase* as of May 27, 2016) that have the highest probability of eventual clinical success as measured by the tensor factorization model. The full list is available in the supplement. For illustrative purposes, we list a related indication approved for assets for each target

As an example, interleukin 6 (IL6) is an approved drug target for giant lymph node hyperplasia (Table 3). Our results suggest that the current trials for psoriatic arthritis, which includes a Phase IIb trial of a monoclonal antibody against this protein [38], have a greater than random chance of success. Psoriatic arthritis is chronic inflammatory arthritis that is associated with psoriasis and thus somewhat related to the successful indication for IL6, a cytokine with a wide variety of biological functions. It induces the acute phase response and is involved in the final differentiation of B-cells into Ig-secreting cells in lymphocyte and monocyte differentiation. It acts on B-cells, T-cells, hepatocytes, hematopoietic progenitor cells and is required for the generation of T(H)17 cells. It also acts as a myokine and is discharged into the bloodstream after muscle contraction and acts to increase the breakdown of fats and to improve insulin resistance [39]. Genetic polymorphism of IL6 has been shown to be significantly associated with a form of psoriatic arthritis [40], and serum IL6 is considered as a biomarker for assessing disease activity in patients with psoriasis, as well as for predicting responsiveness of joint symptoms to biologic treatment [41].

Another target of interest is angiotensin II receptor type 1 (AGTR1), an important effector controlling blood pressure and volume in the cardiovascular system. It has been approved for many cardiovascular indications such as heart failure, myocardial infarction, and hypertension. The predicted indication for AGTR1 is hypercholesterolemia, also known as high cholesterol. AGTR1 antagonism improves hypercholesterolemia-associated endothelial dysfunction [42] and attenuates the inflammatory and thrombogenic responses to hypercholesterolemia in venules [43]. Significant association of AGTR1 polymorphism with hypercholesterolemia was also observed in hypertension patients [44].

## Discussion

In this paper, we focused on the problem of predicting clinically promising therapeutic hypotheses using associative knowledge of targets and indications. We compared tensor factorization with other traditional machine learning methods in a variety of benchmarking experiments and identified two interesting findings from the evaluation of this method: 1) the latent factors learned from the model align with known biological relationships among three human disease areas, and 2) the method can be applied to different scenarios of drug discovery and achieves competitive prediction performance.

However, there are some limitations worth discussing before deploying tensor factorization to propose novel target-indication hypotheses. First, the model relies on the available compilation of evidence sources. Open Targets provided us with a good foundation, but clearly, more sources could be gathered. Second, we treated every clinical failure equally. Our preliminary analysis has shown that some target-indications pairs have been tried multiple times and are still being pursued clinically, while some failed only once and were never tested again. Although the probabilistic framework of the model can potentially mitigate this problem, the model does not explicitly differentiate definitive failures from those that have not been thoroughly explored and may become successful drugs in the future. Lastly, we only applied the technique to a dataset of targets and indications with at least one clinical outcome; thus, the application as benchmarked here is constrained to applying approved drug targets to new indications. The methodology, however, can be expanded to any target and any indication so long as their evidence is encoded in the data. Such an application may result in the identification of novel target-indication hypotheses with a high predicted probability of being successfully translated into medicines.

Computational prediction of drug targets has been widely studied in the context of predicting disease-associated genes [14–16, 45–47]. These disease-associated genes can facilitate the discovery of drug targets by narrowing down the search space of potential targets. The prediction performance (precision) of the models varies from 0.5 to 0.9 depending on the methods and data used in the studies. Many related studies design models to infer novel associations by leveraging similarity information between biological entities and biomolecular network information encoded in a protein-protein interaction database [47, 48]. One example is FASCINATE [17], which is able to infer cross-layer dependencies on multi-layered biological networks. This method can be used for this problem by collapsing all evidence and augmenting the data with disease similarity information.

## Conclusion

In this work, we evaluated a machine learning technique called tensor factorization on the problem of predicting clinical outcomes of therapeutic hypotheses using existing association evidence between drug targets and disease indications. We illustrate that the method can achieve equal or better prediction performance compared with a variety of baseline models across three scenarios of drug discovery, and the learned model can capture the known biological mechanism of human diseases. Furthermore, we demonstrated an application of the method to predict outcomes of trials on novel indications of approved drug targets. Future work includes expanding this method to targets and indications that previously have never been clinically tested and proposing novel target-indication hypotheses that can be developed into medicines with predicted high probabilities of success.

## Additional files

Additional file 1: Supplementary material. (PDF 490 kb)

Additional file 2: Target-indication association evidence data file. (ZIP 227 kb)

Additional file 3: Target-only attribute data file. (ZIP 5 kb)

Additional file 4: Results of benchmark experiments. (XLSX 11 kb)

## Abbreviations

AUPRC: Area Under Precision Recall Curve
AUROC: Area Under Receiver Operator Curve
BTF: Bayesian Tensor Factorization
GBM: Gradient Boosting Machine
LR: Logistic Regression
MeSH: Medical Subject Heading
MF: Matrix Factorization
RF: Random Forest
