# Supplementary material for: Predicting clinically promising therapeutic hypotheses using tensor factorization
Source: BMC Bioinformatics. 2019 Feb 8;20:69. doi: 10.1186/s12859-019-2664-1 (PMC6368709; doi:10.1186/s12859-019-2664-1)
Supplement: Supplementary file 1 — Supplementary material. (PDF 490 kb) [file 12859_2019_2664_MOESM1_ESM.pdf]

SUPPLEMENTARY MATERIAL

Determine the number of latent factors

In the Bayesian tensor factorization method, we need to specify the number of latent factors to represent each mode of the decomposed tensor in a low-dimensional space. Inspired by automatic relevance determination (ARD) [1] and leveraging MCMC Bayesian inference, we propose a heuristic method to determine the number of latent factors. ARD is mainly used to infer relevant features from a large number of input features. The basic idea of ARD is to assign independent zero-mean Gaussian priors on feature weights. The variances of feature weight priors represent the relevance of different input features. If the variance is zero, then those weights are constrained to be zero, and the corresponding input cannot have any effect on the predictions, therefore making it irrelevant. ARD optimizes these variances to discover which inputs are relevant. Similarly, we can treat latent factors in Bayesian tensor factorization method as latent features and use the same idea of ARD to determine the number of relevant latent factors. Specifically, we leverage the approximate distribution generated from MCMC samples and estimate the variance of latent features by fitting the model using a large enough number of factors. If the variance is close to zero, the corresponding latent factor is set to zero. Then the resulting number of factors with non-zero variance is used as the number of factors to re-fit the model. In practice, it is difficult to set a threshold to determine if the variance is close to zero. Here we chose the point before the last large gap or "elbow" appeared in a plot of latent factor's variance in descending order (Figure S1a). The intuition behind this approach is after this point, the latent factors with low variance will no longer preserve the inherent structure in the data and incorporating these factors will only add noise to the final predictions.

As a comparison, we also ran the three cross-validation experiments on the Bayesian tensor factorization model using a series of latent factors. Interestingly, although the performance increases as the number of factors in general (Figure S1b), there exists a local peak around the chosen number of factors determined by the proposed method, especially in the leave one target class out and leave one disease cluster out settings.

De novo disease clustering

The goal of partitioning indications into disease clusters is to obtain a relatively large grouping of indications for leave one out validation such that indications in a group are more similar than the indications that are not in the same group. A simple way is to directly use the hierarchy structure curated in the MeSH (Medical Subject Headings) system. However, there are two problems with this approach. First, it is not uncommon that one MeSH term is assigned to multiple MeSH trees, which brings the problem of not uniquely assigning indications to a group. Second, since the MeSH structure is human-curated, it is possible that some disease relationships are not captured in the structure. Given these problems, we took an approach to derive a disease partitioning *de novo*.

MeSH similarity. We performed clustering of diseases based on a) disease-disease similarity encoded in MeSH structure and b) the disease-disease co-occurrence in literature to capture the similarity information missed in the MeSH structure, and then merged the two results into one partition using consensus clustering. MeSH similarity between any pair of disease terms was calculated using Lin's [2] and Resnik's [3] methods as described in Nelson [4], using the averaged similarity score of the two methods to construct a disease-disease similarity matrix S. Then we performed hierarchical clustering using Ward's method on disease-disease distance matrix, which is defined as 1-S. Then we cut the hierarchy tree at the level where it can yield ten clusters (Figure S2a). Ten is chosen for practical reasons so that the number of clusters for cross-validation is of the same size as the ones used in the standard leave one target class out cross-validation.

Disease co-occurrence. We used the TERMITE platform from SciBite (www.scibite.com/products/termite) to process the scientific literature (April 29, 2016). We recorded the number of disease pairs occurring in the same abstract, and compared these with counts in which the disease appeared with any diseases, converting the resulting overrepresentation P value and odds ratios into a score (0 for random, 1 for highest possible overrepresentation), which we then clustered as in the MeSH case (Figure S2b).

Merged results. We merged the two clustering results into one using consensus clustering. Specifically, each clustering result induces one disease-disease matrix where 1 represents that these two diseases are in the same cluster, 0 otherwise. We took an average of the two disease-disease matrices induced by the two clustering results, performed hierarchical clustering on the resulting disease-disease matrix using Ward's method and cut the hierarchical tree at the level corresponding to ten clusters (Figure S2c). The ten clusters are named after by the most abundant MeSH root term in each cluster. We further grouped cluster 8 and cluster 9 into one cluster since most of the diseases in these two clusters are infectious diseases. As neoplasm diseases (cluster 3) were not included in this paper, the final number of diseases clusters for the leave-one-out-validation is eight.

Reference

1. MacKay DJ: **A practical Bayesian framework for backpropagation networks**. *Neural Computation* 1992, **4**(3):448-472.
2. Lin D: **An information-theoretic definition of similarity**. In: *Proceedings of the 30th international conference on machine learning: 1998*. 296-304.
3. Resnik P: **Semantic similarity in a taxonomy: An information-based measure and its application to problems of ambiguity in natural language**. *J Artif Intell Res* 1999, **11**:95-130.
4. Nelson MR, Tipney H, Painter JL, Shen J, Nicoletti P, Shen Y, Floratos A, Sham PC, Li MJ, Wang J: **The support of human genetic evidence for approved drug indications**. *Nature Genetics* 2015, **47**(8):856.

Figures

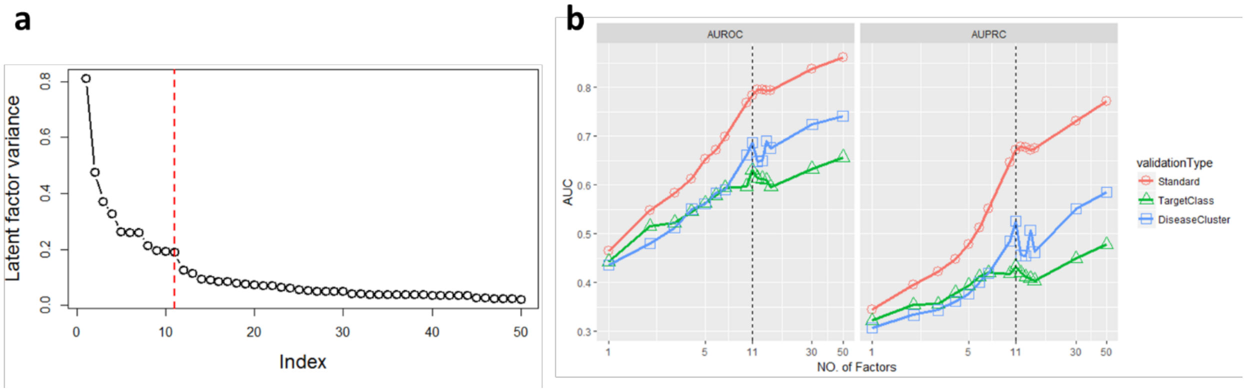

Figure S1. Determine the number of latent factors when fitting BTF models. a) The variance of latent factors when fitting a BTF model to the dataset. b) Prediction performance of BTF model in three validation schemes across a range of latent factors using all available association scores. Dotted vertical lines indicate the number of latent factors chosen based on the last large variance drop.

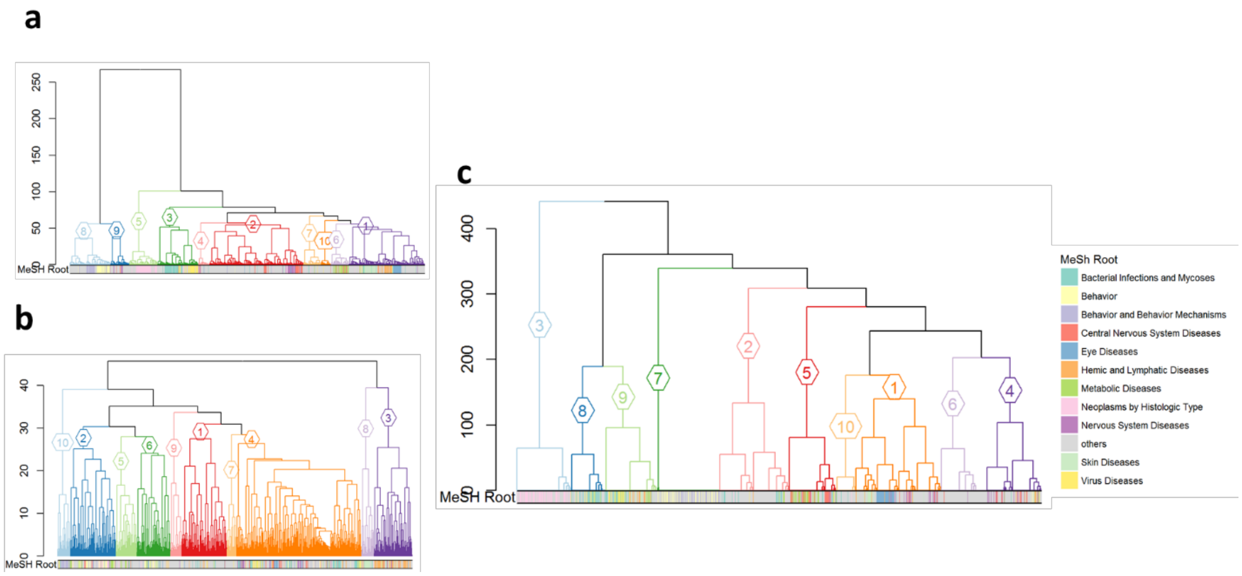

Figure S2. Disease clustering based on disease similarity derived from MeSH and literature mining. Hierarchical clustering of diseases based on similarity derived from a) MeSH structure and b) literature mining. c) Consensus hierarchical clustering of diseases after merging the results from a) and b).

Tables

Table S1. Clinical outcome statistics of target-indication pairs (TIP) grouped by target classes.

Target Class	# of targets	Succeeded TIPs	Clinical Failed TIPs	Total TIPs (%)
Enzyme_other	181	402	727	1129 (18)
Extracellular Ligand	77	73	308	381 (6)
7TM	149	688	1048	1736 (28)
Protease	66	116	223	339 (6)
Transcriptional Factor	41	164	234	398 (6)
Transporter	29	105	116	221 (4)
Other	84	62	220	282 (5)
Receptor_other	103	192	475	667 (11)
Kinase	82	47	311	358 (6)
Ion Channel	63	227	402	629 (10)

Table S2. Clinical outcome statistics of target-indication pairs (TIP) grouped by disease clusters.

Disease Cluster	# of indications	Succeeded TIPs	Clinical Failed TIPs	Total TIPs (%)
Infectious Diseases	126	345	994	1339 (22)
Metabolic Diseases	94	253	476	729 (12)
Cardiovascular Diseases	85	364	628	992 (16)
Musculoskeletal Diseases	71	247	477	724 (12)
GI & Urologic Diseases	70	283	449	732 (12)
Neurological Diseases	81	315	614	929 (15)
Oral Diseases	12	29	42	71 (1)
Mental Processes & Pain	23	228	359	587 (10)

Table S3 63 High Scoring Pairs of Interest from TF Model. New indications of approved targets in clinical trials (Phase* as of May 27, 2016) that have the highest probability of eventual clinical success as measured by the tensor factorization model.

Target	High Scoring Indication in Clinical Pipeline (Phase*)	Prediction Score
AGT	Hepatorenal Syndrome (II)	1.303
IL2RA	Autoimmune Diseases (I)	1.153
SERPINC1	Hemophilia A (II)	1.015
PTGS2	Polyuria (II)	1.004
CYP3A4	Hepatitis C (II)	1.004
PTGS1	Polyuria (II)	1.004
ADRA1A	Polyuria (II)	0.955
IMPDH1	Nephrotic Syndrome (III)	0.948
IL6	Arthritis, Psoriatic (II)	0.941
ADRB2	Surgical Procedures, Operative (II)	0.926
PTGS2	Herpes Simplex (II)	0.914
SERPINC1	Hemophilia B (II)	0.897
CSF3	Menstruation Disturbances (I)	0.881
HTR1D	Sleep Initiation and Maintenance Disorders (II)	0.854
SLC6A2	Surgical Procedures, Operative (III)	0.846

SLC6A4	Surgical Procedures, Operative (III)	0.842
ADRB2	Hypoglycemia (I)	0.841
RYR1	Muscular Dystrophy, Duchenne (I)	0.829
FGFR3	Depressive Disorder, Major (II)	0.828
IFNAR2	Polycythemia Vera (III)	0.828
SLC5A2	Hypertension (III)	0.826
ADRB1	Cachexia (II)	0.815
PTGS2	Cachexia (II)	0.807
OPRM1	Depressive Disorder, Major (III)	0.796
TNFSF11	Hypercalcemia (II)	0.793
OPRK1	Dermatitis, Atopic (II)	0.790
ADRA2A	Surgical Procedures, Operative (I)	0.773
NR3C1	Liver Cirrhosis, Biliary (III)	0.771
ADRB2	Myocardial Infarction (III)	0.769
FGFR3	Multiple Sclerosis, Chronic Progressive (III)	0.757
SRD5A2	Acne Vulgaris (II)	0.752
CASR	Hematopoietic Stem Cell Mobilization (II)	0.746
ADRB2	Cachexia (II)	0.741
HRH1	Opioid-Related Disorders (III)	0.731
OPRK1	Depressive Disorder, Major (III)	0.730
ABCC8	Glucose Intolerance (III)	0.728
PDE3A	Hypercholesterolemia (I)	0.721
OPRD1	Pseudobulbar Palsy (I)	0.719
PTGS1	Opioid-Related Disorders (III)	0.716
OXTR	Atrophic Vaginitis (II)	0.708
ADRB1	Diabetic Foot (II)	0.706
KCNJ11	Glucose Intolerance (III)	0.705
PTGS2	Opioid-Related Disorders (III)	0.696
AGTR1	Hypercholesterolemia (III)	0.691
SLC6A4	Respiratory Insufficiency (II)	0.690
PLG	Retinal Vein Occlusion (I)	0.688
CALCR	Cerebral Palsy (II)	0.682
F10	Cerebral Hemorrhage (I)	0.681
CYP3A4	Herpesviridae Infections (III)	0.677
SOST	Hypophosphatasia (II)	0.671
FKBP1A	Dermatitis, Contact (II)	0.671
CNR1	Cerebral Palsy (III)	0.667
IL6	Waldenstrom Macroglobulinemia (I)	0.655
IL2	Behcet Syndrome (II)	0.650
VDR	Cachexia (I)	0.644
OPRM1	Schizophrenia (III)	0.638

NR3C1	Scleritis (I)	0.638
ADRA2A	Pain, Postoperative (II)	0.637
NR1H4	Liver Cirrhosis (I)	0.637
SLC6A4	Multiple Sclerosis, Relapsing-Remitting (II)	0.631
CALCR	Hemiplegia (II)	0.628
VDR	Alopecia (I)	0.623
IL6	Vasculitis (III)	0.620
